# Optimization of Chitosan Extraction Process from *Rapana venosa* Egg Capsules Waste Using Experimental Design

**DOI:** 10.3390/ma16020525

**Published:** 2023-01-05

**Authors:** Daniel Dinculescu, Cristiana Luminița Gîjiu, Manuela Rossemary Apetroaei, Raluca Isopescu, Ileana Rău, Verginica Schröder

**Affiliations:** 1 Faculty of Chemical Engineering and Biotechnologies, University POLITEHNICA of Bucharest, 011061 Bucharest, Romania; 2 Faculty of Navigation and Naval Management, Mircea cel Batran Naval Academy, 900218 Constanta, Romania; 3 Faculty of Pharmacy, Ovidius University of Constanta, 900527 Constanta, Romania

**Keywords:** chitosan oligomers, *Rapana venosa* egg capsules, artificial neural networks, extraction optimization

## Abstract

New green and sustainable sources were chosen to obtain chitosan, an important material, with many applications in different fields. The present study is focused on egg capsules of *Rapana venosa* waste as raw material for chitosan oligomers. As previous studies revealed that chitosan extraction from this material takes place with a low yield, the present research aimed to optimize this step. A 2^2^ experimental plan, with three replicates in the center, was proposed to investigate the influence of NaOH concentration and temperature on the yield extraction. After a primary analysis of the experimental data, a favorable temperature value was selected (90 °C) at which the total dissolution of the egg capsules was obtained. Then, at this temperature, the experimental plan was extended exploring the influence of the NaOH concentration on three levels (5, 6, and 7%) and the extraction duration on two levels (60 and 85 min). Based on all experimental data, a neural model was obtained and validated. The neural model was used to maximize the yield, applying Genetic Algorithm (GA) implemented in Matlab^®^. The resulting optimal solution is: NaOH concentration 6.47%, temperature 90 °C, duration 120 min, with a yield value of 7.05%.

## 1. Introduction

Chitin and chitosan are biopolymers mainly of marine origin, considered materials support, which are met in organisms such as arthropods, mollusks, worms, coelenterate, and sponges.

Chitin is found particularly in the arthropod’s marine exoskeleton, in the mollusks’ shells, in the cephalopod’s structure (squids, octopuses, etc.) or in the cell walls of some fungi [1], while chitosan can be found in fungi, molds, yeasts, algae [2] or in other sources of rudimentary marine invertebrates.

The studies carried-out in the last fifty years have shown that the waste of crustaceans represents a rich source of products with added value, such as: chitosan, proteins, pigments, whose percentage in their composition, varies with the type of crustacean species and the harvest season [3,4,5].

Chitosan is the only alkaline polysaccharide in nature, is nontoxic, odorless, biocompatible and biodegradable [6]. Depending on their main characteristics (molar mass Mw and deacetylation degree DD), chitosan is made-up from different units of glucosamine and N-acetylglucosamine, or glucosamine only. In addition, the molar mass value is the criterion upon which the chitosan can be classified into high molar mass chitosan (HMw), medium molar mass (MMw), low molar mass (LMw) chitosan, and oligochitosan. Even if the borders between these chitosan types are fluidic, the “oligochitosan” term can be used only for chitosan molecules with fewer than 100 glucosamine units, or molar mass MW less than 16 kDa [4]. On the other side the literature data showed that the oligochitosan with degrees of polymerization (DPs) smaller than 20 and an average molecular weight less than 3.9 kDa are called chitosan oligomers (COs). COs are obtained by depolymerization of chitin or chitosan using acid hydrolysis, hydrolysis by physical methods and enzymatic degradation [5,6,7]. Due to the fact that COs are composed mainly of short chains they tend to change their conformation and form low-viscosity aqueous solutions, which may facilitate the interaction with other polyanions [8].

Oligochitosan is known to have several advantages over HMW and LMW chitosans in biomedical and industrial applications. In particular, due to its lower viscosity and higher compatibility with many additives, the applications of oligochitosan and COs have no undesirable impact on the physicochemical properties of consumer products. Additionally, oligochitosan has some advantages over HMW and LMW chitosan in its biological activity and biomedical applications [4].

Corroborating different literature data [9,10,11,12,13], the presence of particular chitosan or chitin types depends on the degree of evolution of the marine organism. Thus, primary forms, less evolved, without mineralization could be important sources of COs, while developed forms possess chitin, which in its hard-crystalline form, provides support and strength to marine organisms, resulting in the mineralized shell or shells forms. Taking into consideration this information, there is the possibility to find an eco-friendly marine source, susceptible to contain chitosan, a more economical, non-perishable, and clean source as an alternative to the crustacean wastes.

To the best of our knowledge there are few studies on chitosan obtained from such capsules. Our previous studies [11,14] showed the presence of COs in the egg capsules of *Rapana venosa* structure, together with other components, such as proteins and insoluble compounds. COs have a particular importance tacking into consideration their antimicrobial properties and, in this respect, the present study aimed to optimize the process of obtaining COs from *Rapana venosa* egg capsules, by using neural network modelling of the extraction process and GA to obtain the best solution in terms of chitosan yield. Process optimization attempts for chitosan extraction from various sources mention experimental design methods and statistical modelling coupled with nonlinear optimization techniques [15,16,17]. It is important to note that these capsules are either organic agglomerations found on the beach, after storms, or they are the remains obtained from harvesting and cleaning snails intended for consumption and their processing does not represent a threat to the marine environment.

## 2. Materials and Methods

### 2.1. Materials

**Figure 1:**
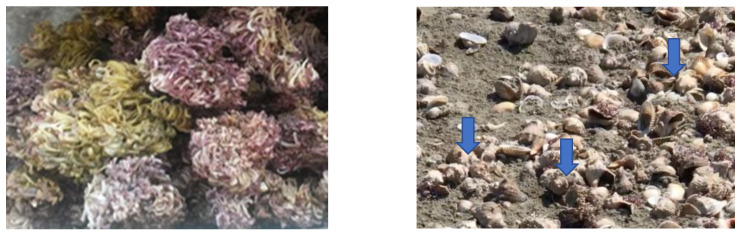
*R. venosa* egg capsules waste.

Chitosan was extracted and identified from *Rapana venosa* egg capsules (Figure 1). The egg capsules free of egg mass, were collected (summer-autumn 2021), from the northern area of Navodari (44°19.3′ North–028°41.7′ East).

The reagents used in the extraction procedure were natrium hydroxide solutions (4%, 5%, 6%, 7%) prepared from NaOH pellets purchased from ChimReactiv S.R.L. (Bucharest, Romania), with purity higher than 99.3%. The acetic acid used for chitosan solubilization, as well as ethanol and acetone (p.a.) were purchased from Sigma-Aldrich (Merck KGaA, Darmstadt, Germany).

### 2.2. Chitosan Extraction Procedure

**Figure 2:**
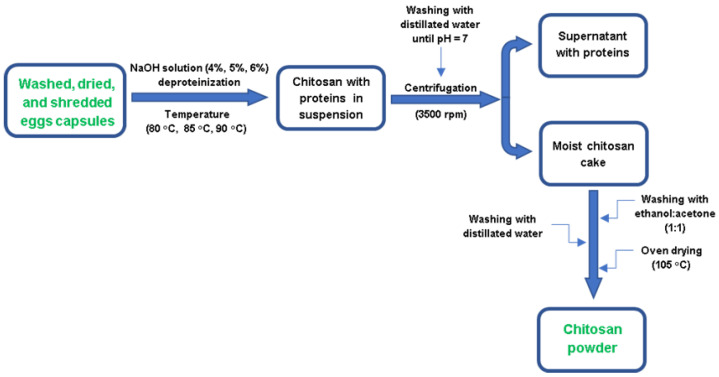
Chitosan obtaining process from *Rapana venosa* egg capsules.

The experimental study used for chitosan extraction from capsule waste the procedure presented schematically in Figure 2. The chitosan extraction obtaining consisted of suspending the cleaned and crushed capsules in alkaline NaOH solutions, of different concentrations (4%, 5% or 6%), in a constant ratio capsule mass: NaOH solution volume = 1:40 (m/v), working time of 65, 85, and 120 min, at different deproteinization temperatures (80 °C, 85 °C or 90 °C), under constant stirring (500 rpm).

After the time chosen for extraction has elapsed, the alkaline suspension resulted was centrifuged at 3500 rpm rotations, in order to separate the precipitated chitosan from the alkaline supernatant loaded with the lipids and proteins that made up the capsular walls. The washing of the chitosan pellets was carried out by successive washings with distilled water until a pH = 7 of the last washing water was obtained.

In order to remove residues, the obtained chitosan was washed with a 1:1 mixture of ethyl alcohol and acetone, in the ratio of wet chitosan mass: solvent mixture volume = 1:10 (m/v). The organic solvents were removed by centrifugation and repeated washings with distilled water. At the end, the obtained wet chitosan cake was dried in an oven at a temperature of 105 °C.

### 2.3. Analytical Methods

#### 2.3.1. Microscopic Analysis

The extracted chitosan from *Rapana Venosa* egg capsules was first analyzed and identified by fluorescence microscopy. Microscopic observations were carried-out by using an optical microscope OPTIKA B—353LD2 (blue filter: λ_ex_ = 450–490 nm; λ_em_ = 515–520 nm), equipped with a camera OPTIKAM, with software Optika Vision Pro, Version 2.7, with (OPTIKA S.R.L., Ponteranica, BG, Italy) or acquisition and data processing.

#### 2.3.2. Determination of Deacetylation Degree

Determination of deacetylation degree (DD) is one of the most important properties of the chitosan chemical characterization, which can influence its performance in many applications.

To determine the DD, the potentiometric titration [15] was taken into consideration, and the value of DD (%) was calculated according to [18] study (Equations (1) and (2)).

(1) $$DD\;(\%)=\frac{203\cdot Q}{1+42\cdot Q}$$

(2) $$Q=\frac{c_{M}\cdot \Delta V}{m}$$

where: c_M_—molar concentration of NaOH solution, used for titration (mol/L, in the present study: 0.1 mol/L);

ΔV—volume difference of NaOH added corresponding to the two inflection points (L);

m—mass of analyzed chitosan (g).

#### 2.3.3. Molar Mass

The method used to determine the molar mass was based on intrinsic viscosity determination of the chitosan dissolved in dilute acidic solutions. The measurements were carried out using a capillary viscometer (Ostwald type), according to Pădurețu et al. [15].

This method is based on the relationship between the viscosimetric-average molar mass, Mv (g/mol) and the intrinsic viscosity, [η] (mL/g) of the chitosan solution given by the well-known Mark–Houwink–Sakurada Equation (3), where K and α are constants which depend on the nature of the solvent, temperature, and chemical structure of the polymer (K = 13.8 × 10^−3^ mL/g, α = 0.85).

(3) $$\left[\eta \right]=K\cdot M_{v}^{\alpha }$$

## 3. Results and Discussion

### 3.1. Optimization of the Extraction Process

The research started with an experimental plan 2^2^ with three replicates in the center for the evaluation of the experimental error (variance 0.33). Two factors were considered in these steps: the concentration of NaOH and the temperature, while the duration was maintained constant at 120 min.

The obtained data are presented in Table 1.

The primary analysis of the results showed that not all the investigated conditions led to desired results, experimental point 1 corresponding to the concentration and temperature at minimum values even leading to the non-dissolution of 50% of the capsules. As a result, this point was excluded, and the experimental plan was completed with another 12 experiments carried out at a temperature of 90 °C, as a highest value of temperature, proved favorable for the total dissolution of the capsules (Table 1). A new experimental plan was proposed considering the factors the NaOH concentration and the process duration. A variable number of levels was chosen. For the process duration two levels were considered, while three levels were defined for the NaOH concentration in order to better evaluate its influence upon the extraction yield. These experiments were replicated twice at each point. The data are summarized in Table 2.

### 3.2. Optimizing the Extraction Yield Using Neural Networks

The data used for modeling are taken from Table 1 and Table 2, omitting the 50% undissolved samples, but keeping the partially dissolved and gelatinized samples, resulting in a total of 18 samples.

**Figure 3:**
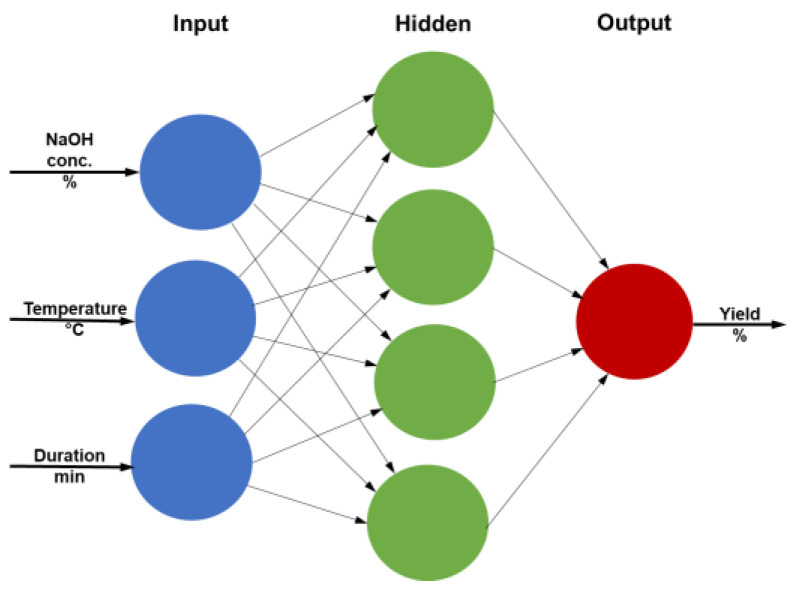
ANN architecture.

The evaluation of process yield as a function of NaOH solution concentration, temperature and duration was based on artificial neural networks (ANN) building. ANN belongs to the stochastic modelling and, even if they apparently are similar to regression, a totally different concept is applied to explain the influence of factors (inputs) upon the dependent variable (output). The process features are captured during a learning stage (training and testing) based of which the network will be capable to reproduce the process. During the learning stage, the sum of errors between the measured values of the output and the estimated ones is minimized according to a selected learning rule and optimization method. The resulting model is a “gray box” that reflects the relation between the inputs and outputs, even if a direct mathematical relation cannot be written. For the present study, a multilayer ANN was proposed (Figure 3) with three neurons in the input layer standing for the NaOH concentration, temperature, duration, four neurons in the hidden layer and one output neuron representing the extraction yield.

The network was trained and tested in Matlab (R2015a) using: Activation function: sigmoid; Learning Rule: Backpropagation of errors and Levenberg–Marquardt optimization algorithm implemented in the “trainlm” function, and population size 100.

In order to avoid overfitting, 70% of the data were used for training and 30% for testing and validation.

A first neural model obtained with the initial data achieved a correlation of the data with a coefficient of determination R^2^ = 0.92.

The optimization of the extraction process, in the sense of maximizing the yield, with the use of the neural model obtained at this stage, was carried out with the GA algorithm implemented in Matlab.

The resulting optimal solution is: NaOH concentration 6.7%, temperature 89 °C, duration 120 min, with a yield value of 5.75%.

To test the neural model, two experimental points were proposed in the «proximity» of this first estimate of the optimum (Table 3).

**Figure 4:**
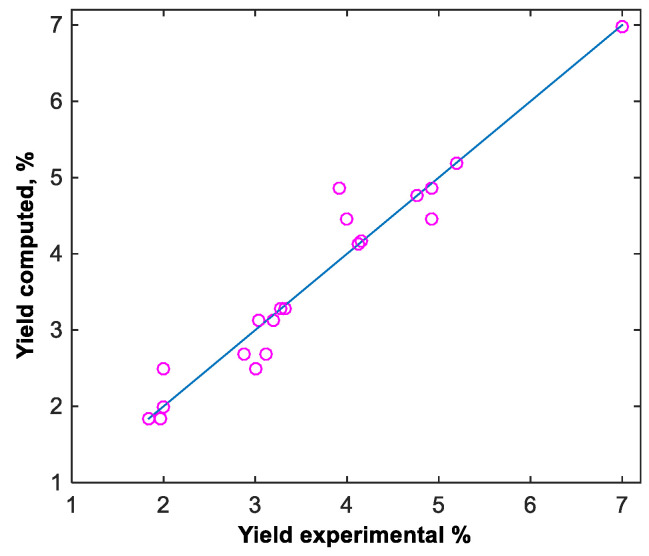
Parity plot for the experimental and computed data.

In order to obtain a better model, these two new points were added to the data set and a new improved neural model with the same structure, based on 20 experimental measurements, was obtained, for which R^2^ = 0.94 (Figure 4).

**Figure 5:**
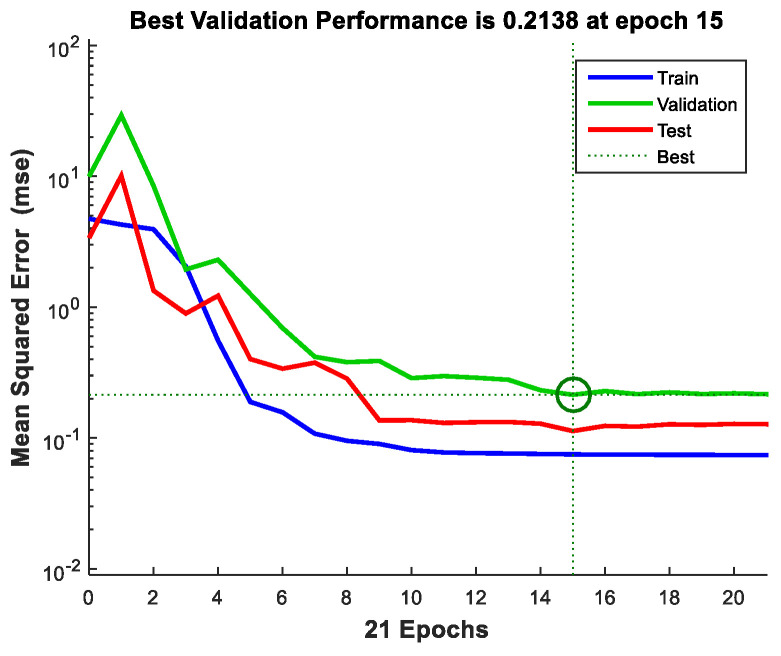
Evolution of the error in approximation of experimental data, during the learning process.

From the point of view of how the network captured the characteristics of the process, the convergence variation in the training, validation, and testing stages is analyzed, by identifying the minimum errors between the measured and estimated yield values, during the learning cycles (Figure 5).

**Figure 6:**
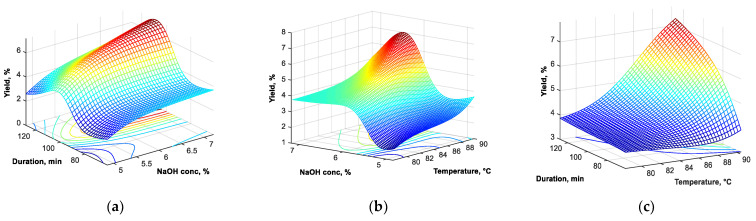
Yield variation for 90 °C temperature (**a**), 120 min working time (**b**) and 6.5% NaOH concentration (**c**).

Graphic representations of the yield variation in the investigated field showed that both the temperature and the working time must be at the maximum investigated values (90 °C and 120 min), while the optimal concentration of NaOH is close to the value of 6.5%, the increase in this concentration up to 7% or above this value not being favorable (Figure 6).

The optimum identified using the genetic algorithm suggested the operating conditions: NaOH concentration 6.47%, temperature 90 °C, duration 120 min at which the estimated yield is 7.05%.

Both the operating conditions and the yield value are very close to the values of experimental point two in the set of validation experiments; therefore, these conditions no longer require experimental validation, and the set of operating parameters [6.5 90 120] can be considered as optimal in the investigated field.

### 3.3. Chitosan Characterization

#### 3.3.1. Fluorescence Microscopy

**Figure 7:**
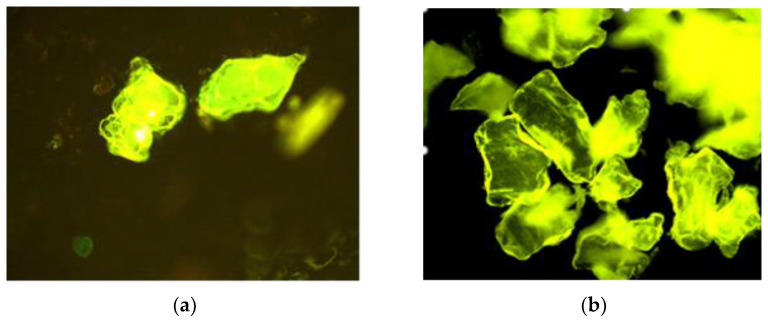
Fluorescent emissions (epifluorescence microscopy (×100)) of: (**a**) chitosan powder extracted from capsules; (**b**) commercial chitosan powder.

Cellulose, lignin, chitosan and collagen are important structural compounds existing in many organisms’ bodies. These biopolymers emit fluorescent radiation and these natural emissions are due to the presence, in various compartments of cell, of fluorescent compounds which emit radiations in the spectrum of visible light when they are excited with UV light [19]. The chitosan powder obtained by chemical extraction from the *Rapana venosa* egg capsules in alkaline dilute solutions was analyzed by the epifluorescence microscope, and its fluorescent emissions were compared with those emitted by the powder of a commercial chitosan (Figure 7a,b).

Comparing Figure 7a with Figure 7b, it can be observed that the two chitosan powders fluorescent emissions subjected to microscopic observations are similar, although the commercial chitosan is of a different origin.

#### 3.3.2. Deacetylation Degree (DD) and Molar Mass (MW)

For the extracted chitosan, the DD values ranged between 74% and 76%. The obtained results were compared with the values of one low molar mass commercial chitosan (product number: 448869, CAS 9012-76-4, purchased from Sigma Aldrich, Darmstadt, Germany), with DD = 95%.

According to our experimental data, the obtained values for the molar mass of the chitosan extracted from egg capsules of *Rapana venosa* snail range between 1.5 and 3 kDa, which classifies this biopolymer into the category of chitosan oligomers (COs) [8,9]. The molar mass of extracted chitosan from egg capsules of *Rapana venosa* (1.5 kDa) was lower than the one obtained for the commercial chitosan (183 kDa—extracted from crab shells). This difference in the molar mass is due to the different sources of raw materials.

## 4. Conclusions

The present study proved that this new waste represented by *Rapana venosa* egg capsules represent an eco-friendly source for chitosan, particularly for chitosan oligomers with a deacetylation degree of 75%. This new source of COs-type chitosan presents, on one hand, the advantage to be obtained at low costs, by valorizing some marine waste, and on the other hand, it could be used as a biocide, in various fields of environmental engineering. By tailoring the extraction parameters, a reasonable yield (around 7%) can be achieved despite the fact that these capsules contain a low content of chitosan. The artificial neural network modelling and the genetic algorithms used in the optimization study revealed that the optimal operating parameters are a NaOH concentration of 6.5%, a temperature of 90 °C and an extraction time of 120 min.

## Figures and Tables

**Table 1:** Yields values for the initial 2^2^ experimental plan, considering an extraction time of 120 min.

Exp. No.	NaOH Conc. (%)	Temperature (°C)	Yield (%)	Observations Deproteinization Time = 120 min m_egg_:vol NaOH = 1/40
1	4	80	1.72	At the end, about 50% egg capsules did not dissolve
2	6	80	4.16	At the end, part of the capsules remained undissolved and were found in the centrifuged filtrate
3	4	90	4.76	At the end, all the capsules were dissolved
4	6	90	5.20	At the end, all the capsules were dissolved
5	5	85	3.00	At the end, when filtering the obtained suspension, a small number of capsules remained in the form of gelatine (partially dissolved)
6	5	85	2.00	
7	5	85	2.00	

**Table 2:** Yields values for the supplementary 2^2^ experimental plan for the deproteinization temperature of 90 °C.

	Duration (min.)	60	85
NaOH Conc., (%)			
**5**		1.96	3.04
		1.84	3.20
**6**		3.32	4.92
		3.28	3.92
**7**		2.88	4.00
		3.12	4.92

**Table 3:** New experimental points, and the yield values.

Exp. No.	NaOH Conc. (%)	Temperature (°C)	Duration (min)	Yield (%)	
				Estimated by the Preliminary Model	Experimental
1	7	85	120	4.15	4.13
2	6.5	90	120	6.18	7.00

## Data Availability

Not applicable.
